# Supplementary material for: Are carbon emissions trading and green financial instruments synergistic? -Comprehensive quantitative research based on content analysis
Source: PLoS One. 2024 Mar 7;19(3):e0298601. doi: 10.1371/journal.pone.0298601 (PMC10919601; doi:10.1371/journal.pone.0298601)
Supplement: S1 Data — (ZIP) [file pone.0298601.s001.zip › Data/地级市Z_F工作报告.zip!/╔┬╬≈/╔╠┬σ╩╨/└┤╫╘╣1⁄2╓┌║┼í╛┬φ┐╦╩2╛▌═°í┐_2014.docx]

各位代表：

现在，我代表市人民政府向大会作工作报告，请予审议，并请市政协委员和其他列席人员提出意见。

一、2013年工作回顾

2013年是我市深化发展、提质发展的重要一年。这一年，面对错综复杂的经济形势和下行压力，在省委、省政府和市委的正确领导下，全市上下认真贯彻落实党的十八大精神，紧紧围绕建设幸福商洛的总目标，牢牢把握“稳中求进”的总基调，以创新破难题，以实干促赶超，全市经济稳中有进、稳中趋优，较好地完成了市三届人大三次会议确定的目标任务。

——国民经济平稳增长。实现生产总值510.88亿元、增长12.6%；财政总收入39.7亿元，其中地方财政收入25.68亿元，分别增长17.7%和19.4%。

——发展质量持续提升。三次产业结构为17.3∶50.3∶32.4，与上年相比一产下降0.8个百分点，二产提高2.3个百分点，三产回落1.5个百分点；非税收入比重较上年下降3个百分点；非公有制经济增加值占GDP的比重达到50.92%。

——发展后劲显著增强。全社会固定资产投资496.16亿元、增长26.7%，其中民间投资260.7亿元、增长40.4%；招商引资到位资金309亿元、增长36.39%；社会消费品零售总额121.82亿元、增长13.6%。

——人民生活不断改善。城镇居民人均可支配收入22257元，农民人均纯收入6223元，分别增长11.3%和14.7%；城镇登记失业率控制在3.4%以内；居民消费价格指数控制在2.6%以内。

——社会事业全面发展。高考二本以上上线率47.58%，连续六年位居全省第一；新农合参合率103.8%，连续十年位居全省第一；文化产业增加值5.45亿元、增长29%；人口自增率3.02‰。

——社会大局和谐稳定。社会治安满意率、平安创建知晓率和公众安全感综合指数均位居全省前列，进京非访人次和赴省集访批次分别下降45%和59%，安全生产事故起数和死亡人数分别下降39.5%和36.7%。

一年来，我们重点抓了以下十个方面的工作：

(一)持续加大重点项目建设和投资力度。认真落实《陕西省重点项目推进办法》，着力在优化投资结构、发挥民间资本作用、提高资金使用效益、完善评价考核体系等方面下工夫，争取“一县一产业”和陕南循环发展资金1.54亿元，落实项目建设用地1.74万亩，150个市级重点项目建设进展良好。一批具有引领作用的重大项目加快推进，柞水同兴轧钢50万吨棒材、商南国际彩印包装产业园等32个项目建成或投产；西康铁路复线建成运营，西合铁路复线建设进展顺利，改造提升干线公路102公里，丹江流域综合治理启动实施。扎实推进重大项目前期工作，落实重大项目前期费1.3亿元，48个重大前期项目基本具备开工条件。

(二)把保工业增长摆在更加突出位置。围绕工业增产促销提效，争取和落实工业专项扶持资金8235.5万元，全年新增产能50亿元，主要工业品产销率达到91%；加大骨干企业扶持力度，新增规模以上工业企业32户，实现规模以上工业增加值145.2亿元、增长27%。出台了支持小微企业健康发展的若干意见，全年新增私营企业726户、个体经营户8693户。加快推进商丹园区创新发展，山阳中村等3个园区新确定为省级重点县域工业集中区，提升了县域工业集中区管理规格，新增入园企业52户。积极培育高端饮用水产业，组建了秦岭健康水产业研发中心，富氢水开发项目进展顺利。加强企业创新能力建设，全年申请专利985件、技术合同成交额4376.38万元，分别增长75.5%和26.67%，全市七区县通过国家科技进步考核。

(三)千方百计促进农业增效农民增收。以建设现代农业示范园区为引领，落实市级农业产业化专项资金1500万元，加快农业特色产业基地建设，培育现代农业园区57个，其中新认定省级园区4个。农业实现“六连丰”，粮食产量65.07万吨，核桃等优势产业加快发展，肉、蛋等主要农产品产销两旺。大力培育新型农业经营主体，新认定省级龙头企业8户，发展专业大户86户。加强农业品牌建设和商标培育，新认定“三品”产地43个，认证“三品”产品60个，注册农产品商标26件。加强农业综合生产能力建设，新修加固堤防227公里，新建基本农田7.38万亩，解决农村饮水困难24.15万人。

(四)加快发展旅游等现代服务业。坚持大型景区游和乡村休闲游相结合，着力发展全域旅游，积极促进旅游联盟。山阳月亮洞、商南闯王寨等景区对外开放，天竺山、牛背梁创建为国家4A景区，木王等7个景区创建为国家3A景区。全面启动“秦岭美丽乡村”创建活动，对商南任家沟等16个村进行了首批命名。我市成为全省首个省级旅游服务标准化城市，跻身“全国百强旅游城市”和“最具魅力旅游城市”。举办了2013中国秦岭生态旅游节，全年接待游客2764.5万人次，综合收入135.4亿元，分别增长20.8%和32.1%。大力发展现代服务业，培育限额以上消费企业27家，汽车等大宗消费增长55.1%。

(五)着力加快城乡一体化发展。深入推进“城市建设管理年”活动，挂牌成立了市测绘地理信息局和城市规划勘测院，城市规划、建设和管理水平进一步提高。加快推进中心城市建设，编制了“一体两翼”地区发展规划，出台了市区共建中心城市实施意见，“一体两翼”中心城市建设投资25.94亿元，投资规模与上年相比实现翻番。环城北路、西街及莲湖东侧旧城改造等项目取得实质性进展，龟山大道、商鞅大道东段、西商二线连接线跨丹江大桥建成通车。加快县城和重点镇建设，积极推进新型社区建设，全市城镇化率达到45.53%。

(六)大力推进循环发展和环境治理。规划的10条循环产业链7条初步建成，20个循环经济标志性工程10个基本建成，大西沟“矿钢一体化”初具雏形，光伏全产业链成为全省典型，我市成为全省首个循环经济试点城市、国家首批循环经济示范创建城市。加强环境治理和生态保护，启动了丹江等流域污染防治三年行动计划，全年实施节能减排项目69个，淘汰铅冶炼等落后产能18万吨，单位GDP能耗下降3.3%，化学需氧量、氨氮、二氧化硫和氮氧化物排放分别削减2.5%、2.2%、2.8%和3.3%；强化对建筑扬尘的监管，全市淘汰黄标车910辆，停用拆改燃煤锅炉215台，市区空气质量达到或好于二级天数达351天。扎实推进生态建设，七区县全部创建为省级园林城市，完成城周绿化3.5万亩，植树造林37.2万亩，治理水土流失806平方公里。 （下转第四版）

(七)持续提升民生保障工作水平。全市民生财政支出117亿元，实施基本建设类民生项目1100个。加快推进保障性住房建设和陕南移民搬迁，建设集中安置点118个，搬迁群众1.6万户、6.08万人，9.28万贫困人口实现脱贫；实行廉租房和公共租赁住房并轨管理，建设保障性住房9984套，竣工4846套，累计入住3.9万户，入住率79.37%。加强农民工返乡创业示范县创建，全市城镇新增就业1.4万人，失业人员再就业6000人，大学生初次就业率达到89.4%，农村劳动力转移就业63.1万人。加快完善社会保障体系，企业退休人员养老金、60岁以上城乡居民基础养老金和城乡居民医疗保险政府补贴标准进一步提高，城乡居民社会养老保险参保率达到99.1%，26.2万城乡低保对象实现动态管理下的应保尽保，为13.9万高龄老人发放补贴1.07亿元，全市新增养老床位3000张。

(八)积极促进基本公共服务均等化。学前教育三年行动计划圆满完成，31所公办幼儿园和39个义务教育标准化项目全部完工；“营养改善计划”全面实施，惠及16.6万名学生；丹凤、柞水通过国家义务教育发展基本均衡县验收，山阳“教育强县”、柞水“双高双普”创建通过省政府验收。县级公立医院改革全面展开，药品“三统一”全面实施，让利患者6200万元。深入实施文化惠民工程，完成16.7万户广播电视户户通工程，建成镇办广播电视标准化服务站136个，为230个行政村和社区配送了文化活动器材，为56个镇办及社区建设了电子阅览室，农家书屋实现了行政村全覆盖。成功举办了商洛市第三届运动会，我市运动员王亚栓夺得全国第十二届运动会3000米障碍赛金牌。

(九)深入推进改革创新和对外开放。统筹推进行政体制改革和事业单位分类改革，理顺了市区县城市管理和住房公积金管理体制，完成了全市食品药品安全监管体制改革。积极推进投融资体制改革，完成了市城投公司内部管理改革，成功发行城投债券15亿元；在整合重组创投公司的基础上，组建了商丹园区建设开发公司；成立了市交通投资建设有限责任公司，对市中小企业担保公司进行了增资扩股。扎实开展“营改增”试点，全面整合了财政专项资金。加快金融系统改革创新步伐，镇安农合行改制为陕南首家农村商业银行，新设小额贷款和融资性担保公司2家，完成增资扩股6家。把招商引资作为扩大开放的首要任务，坚持一把手挂帅、全民抓招商，积极组织和参加各类大型招商活动，引进中兴、维维、人人乐等国内500强企业。全市实际利用外资7010万美元，外贸进出口总额6294.1万美元。

(十)全面加强政府自身建设和社会管理。加快转变政府职能，不断创新服务方式，行政效能持续提升。始终坚持依法行政，深入实施“六五”普法，加快依法行政示范市建设，办理人大代表建议170件、政协委员提案265件。“八项规定”和“三个不增加”的要求全面落实，11个市直部门实现了预算公开，强化“三公”经费和领导干部经济责任审计，全市“三公”经费支出下降8.7%，财政供养人员得到严格控制，全市政府系统查处违法违纪案件165起。全面开展邀请公民走进市政府活动，政府联系群众的渠道进一步拓展。深入推进综治和平安建设，加大安全生产监管力度，深化食品药品安全监管，强化矛盾纠纷排查调处，加强社会治安综合治理，巩固了和谐稳定的大好局面。

与此同时，统计、人防、气象、水文、方志、档案、外事、侨务、民族、宗教等工作取得了新成绩，慈善、通信、供销、无线电、工商联、残疾人、妇女儿童、红十字会、国防动员等各项事业有了新发展。

各位代表，过去的一年，我们谋长远、抓大事，解难题、惠民生，取得了令人欣慰的成绩。这一年，我们首次实现人均GDP突破3000美元，标志着商洛已步入一个新的发展阶段。这些成绩的取得，是省委、省政府和市委正确领导的结果，是市人大、市政协监督支持的结果，是全市上下各条战线共同努力的结果。在此，我代表市人民政府，向全市广大干部群众、驻商部队指战员、武警官兵、公安民警致以崇高的敬意！向大力支持政府工作的各位代表、各位委员和社会各界人士表示衷心的感谢！

在肯定成绩的同时，我们也清醒地看到：贫困人口多、发展基础弱、经济欠发达仍是我们的基本市情，自主创新能力弱、科技成果转化滞后、内生增长动力不足仍是我们的主要矛盾，城市化水平低、产业发展层次低、城乡居民收入低仍是我们的发展短板，土地保障难、资金筹措难、环境保护难仍是我们的最大瓶颈，解决“四风”问题、创新体制机制、提高行政效能仍是我们的艰巨任务。对此，我们将采取得力措施加以解决。

二、2014年工作的基本思路和预期目标

今年是贯彻落实党的十八届三中全会精神、全面深化改革的第一年，也是完成“十二五”规划至关重要的一年。我市经济社会发展面临的总体形势是挑战与机遇并存。我国经济进入增长速度换挡期、结构调整阵痛期和前期刺激政策消化期叠加阶段，不稳定不确定不可持续因素依然较多，但是我国经济长期向好的基本面没有改变，中央全面深化改革、加快推进新型城镇化、共建丝绸之路经济带等重大战略部署，为我们带来了新的发展机遇；经过多年的不懈努力，我市在产业结构调整中形成了一些新的增长点，一批重大基础设施项目显著提升了发展保障能力，我市经济发展处于快速增长区间的基础更加牢固，我们完全有条件、有能力保持经济持续平稳较快发展。

今年政府工作的总体要求是：以邓小平理论、“三个代表”重要思想、科学发展观为指导，全面贯彻落实党的十八大和十八届二中、三中全会以及中央经济工作会、中央城镇化工作会、省委十二届四次全会、市委三届五次全会精神，坚持科学发展、富民强市主题和发展循环经济主线不动摇，牢牢把握“稳中求进、改革创新、提质增效”的总基调，继续实施“三大战略”、“四大带动”，依托大西安、建设大商洛、打造大品牌，重点建设“两大板块”、做强“两大经济”、实施“两大工程”，把改革贯穿于经济社会发展各个领域各个环节，在稳定增长中优化结构，在深化改革中创新发展，在扩大内需中推进开放，在改善民生中促进和谐，着力打造商洛经济升级版，让发展成果更多惠及全市人民。

今年经济社会发展的主要预期目标是：全市生产总值增长12%左右，财政总收入、地方财政收入分别增长11%和13%左右，城乡居民收入分别增长12%和14%，城镇登记失业率控制在4.2%以内，居民消费价格指数控制在4%以内，节能减碳及四项减排指标控制在省上确定的范围以内。

三、2014年的重点工作任务

(一)狠抓项目，夯实基础，确保投资稳定增长

坚定不移地推进重点项目建设，全年实施市级重点项目160个，年度投资350亿元以上，力争固定资产投资增长26%。

一是强力推进市区重大项目建设。坚持在建项目抓进度、新建项目抓开工，集中力量推进市区八大重点工程建设，力争形成更多的实物量和投资量。西街旧城改造完成路网配套设施，年底前安置房主体工程竣工；环城北路7月底前实现路通灯亮，9月底前全面交付使用；312国道商州至丹凤段“二改一”、307省道商州至洛南段“三改二”和商鞅大道西段均完成路基主体工程；市一级客运站基本建成；商洛中学迁建完成征地拆迁，年底前主体工程开工建设；市文化艺术中心完成规划设计和招投标，力争上半年开工建设。

二是加快重大产业和基础设施项目建设。实施100个市级重大产业项目，重点推进商州秦岭生态健康养老城、丹凤恒大产业园、洛南钾长石全产业链开发、商南硅产业园、山阳必康医药产业园、镇安抽水蓄能电站、柞水矿钢一体化等项目建设。积极配合西合铁路复线建设，争取洛卢高速列入省级高速公路网，做好山柞高速、榆商高速华阴至洛南段招商工作；积极推进102省道柞水石镇至石瓮、202省道洛南县城至石门、203省道山阳县城至色河等3条二级公路改建工程。配合做好“西气东输”三线工程建设，加快实施商州-丹凤-商南、洪门河-洛南等“气化商洛”工程。加快电力和通讯基础设施建设，完成新一轮农网升级改造任务。

三是完善重点项目推进和保障机制。严格落实各级领导包抓重大项目建设责任制，继续实行重大项目联席会议和观摩制度，坚持每月召开一次市区重点项目建设现场办公会，强化项目质量评估、动态管理及督查考核，探索建立投资管理的负面清单制度。完善项目用地和资金保障机制，加大闲置土地清理力度，有序推进土地整理和复垦，确保重点项目建设用地需求；充分发挥财政资金的杠杆撬动作用，市财政安排工业园区、现代农业园区、旅游精品景区和新型农村社区等“四区”建设及产业引导资金7000万元；积极争取上级支持和银行贷款，做实城投、交投、中小企业担保、商丹园区建设开发和秦宇等市级融资平台，鼓励和支持区县组建投融资机构，着力解决项目建设资金问题。

四是加强重大项目策划和储备工作。扎实做好地质找矿整装勘查工作，在摸清资源家底的前提下，策划一批矿业开发和循环利用项目；围绕国家投资方向和产业扶持政策，策划一批重大基础设施和发展前景好、带动能力强的产业项目，进一步优化投资结构和项目布局。加快推进西商城际铁路等150个重大前期项目，确保重大项目接替有序、滚动发展。

(二)优化结构，集聚发展，做大做强工业经济

以结构调整为导向，以工业园区为平台，以产业培育为抓手，以骨干企业为依托，以科技创新为支撑，提升工业经济发展质量和效益，力争规模以上工业增加值增长23%。

一是调整优化工业结构。深度开发“金石药果”四大优势资源，加大技术突破、链条延伸和改造升级力度，扶持壮大现代材料、现代中药、绿色食品三大工业主导产业。抓住国家促进光伏产业健康发展的政策机遇，抓好太阳能组件、太阳能光伏发电等项目建设，发展壮大太阳能光伏产业。抓住中东部产业转移的机遇，加快超级电容及电动汽车装备、电子商务及物联网、电子集成器等项目建设，培育发展装备制造业和电子信息产业。立足开发优质的水资源，推进水电镁一体化以及富氢水、小分子水开发等项目，加快发展新能源和高端饮用水产业。

二是提升园区承载能力。按照“规划引领、产城融合、生态同步、配套完善”的思路，支持商丹省级工业园区加快发展，启动建设洛河市级工业园区，完善园区管理、投融资、财税分配等体制机制，着力打造丹江和洛河两大产业长廊。坚持“各具特色、错位发展”的原则，加快推进10个县域工业集中区提档升级，完善创业基地和综合服务中心等配套体系，力争全年新增入园企业40户。

三是支持骨干企业发展。制定支持骨干企业发展的政策措施，培育一批主营收入过10亿元的骨干企业，年内新增规模以上工业企业25家。综合运用税费减免、产品收储、贷款贴息、销售奖励等措施，帮助骨干企业和重点行业释放产能、扩大销售，力争主要工业品产销率保持90%以上。

四是推进企业自主创新。继续深化书记市长进高校进院所活动，强化“政企院”合作，推进“产学研”结合，加强循环产业链重点环节关键技术攻关，加快尾矿综合利用、黄姜深加工和钒材料链条延伸等工程技术中心建设，实施10个重大科技专项，推动科技型企业加快发展。

(三)突出特色，提高效益，大力发展生态农业

认真贯彻中央1号文件精神，以创建秦岭生态农业示范市为抓手，突出建基地、育龙头、创品牌、强基础，全面深化农村改革，加快推进农业现代化，促进农业稳定增效、农民持续增收。

一是加快农业优势特色产业发展。按照“稳粮、扩经、强牧、兴药、优果”的思路，大力推行“一镇一业”、“一村一品”，以园区建设为抓手发展多种形式的适度规模经营，重点发展核桃等五大市级优势产业，加快发展茶叶等十个县域主导产业，培育发展食用菌等十个地方特色农产品。从数量和质量上守住耕地红线，确保粮食总产55万吨左右。强化菜篮子行政首长负责制，新发展设施蔬菜6000亩。

二是着力构建新型农业经营体系。推行“公司+合作社+专业大户”的发展模式，坚持内育和外引相结合，做大做强农业龙头企业，推进农副产品精深加工；支持农民专业合作社发展，扶持一批专业大户和家庭农场，培育壮大新型职业农民队伍，构建以公益性农业服务体系为主体，经营性服务组织为补充，形成一主多元的现代农业服务格局。

三是加强质量安全监管和品牌创建。全面开展省级农产品质量安全监管示范区县创建活动，将农产品质量和食品安全纳入区县政府绩效考核范围，建立食品安全监管和责任追究制度，健全从田间到餐桌的全过程监管体系。做好“三品一标”认证工作，培育一批名牌产品和著名商标，打造“山地农产在商洛”品牌。

四是加快完善农业农村基础设施。加强农田水利、城乡供水和农村道路工程建设，加快实施丹江干流和10条中小河流防洪工程，新修基本农田7万亩，新增灌溉面积2万亩；完成10座病险水库除险加固任务，解决10万人的饮水困难问题；实施130公里农村公路交通扶贫项目，完成100公里县乡公路改造，抓好168公里移民安置点配套路网建设。加强防灾减灾和救灾能力建设，启动建设丹江流域人工影响天气基地。

五是积极推进农村改革各项工作。全面落实强农惠农富农政策，增强农业农村可持续发展能力。做好农村承包地、宅基地、集体建设用地确权登记发证工作，加强土地经营权流转的管理和服务，推动农村产权流转交易公平公正规范运行，积极探索增加农民财产性收入的有效途径。

(四)扩大总量，提升层次，加快发展现代服务业

以旅游提档升级为重点，在做活商圈上求突破，在做旺人气上下工夫，提升服务业发展层次，培育经济发展新优势。

一是促进旅游产业提档升级。提升“秦岭最美是商洛”品牌，积极打造大秦岭旅游核心区。继续抓好大型旅游景区建设，加快商於古道文化旅游景区建设，力争丹凤棣花文化旅游区、山阳天蓬山寨等景区开放运营，持续做好金丝峡5A和柞水溶洞、丹江漂流4A景区创建。深入开展“秦岭美丽乡村”创建活动，推进旅游与农业、文化、小城镇建设融合发展。全面推进旅游标准化区县创建，依托中国秦岭生态旅游节，全方位开展旅游宣传促销，开发特色旅游产品和休闲项目，推进门票经济向综合产业、单体景点向全域旅游转型，实现过境游向目的地游、生态观光游向休闲度假游转变，力争全年接待游客达到3000万人次。

二是培育休闲养老健康产业。抓住国家促进健康服务业发展的政策机遇，依托必康、盘龙、天士力等龙头企业，加强与陕西中医学院和中医研究院的战略合作，策划实施一批休闲、养老、康体、健身产业项目，加快构建集生物医药、保健食品、中医养生、医疗康复为一体的健康产业集群。精心筹办首届“环秦岭公路自行车赛”，培育高端养老和休闲健身两大品牌。

三是推进服务业与相关产业融合发展。加快发展物流、仓储、运输、包装等生产性服务业，引导商贸、餐饮等生活性服务业适应市场要求创新发展。加快推进小额贷款保证保险业务开展，鼓励和推动金融咨询类中介机构发展，完成商南、柞水两县农村信用社改制任务，加快丹凤、山阳两县农村信用社改制工作，支持长安银行网点向区县延伸，推进邮政储蓄银行网点业务向镇村延伸。全年新增贷款30亿元以上，实现各类保费收入10亿元。

四是持续繁荣城乡消费市场。加快实施城乡居民收入倍增计划，完善企业工资集体协商制度，确保农民工工资支付到位，严格落实带薪年休假制度，提升居民消费能力和预期。大力培育文化、休闲、体育、健身和信用消费，多渠道引进各类网络服务商，促进电子商务和信息消费发展。加强消费市场监管，着力优化消费环境，力争社会消费品零售总额增长13%。

(五)统筹城乡，建管并重，加快新型城镇化进程

制定实施全市新型城镇化发展规划，坚持做大城市、做强县城、做精集镇、做美村庄、做好社区，构建科学合理的城镇体系，全市城镇化率提高到47.5%。

一是全力推进“一体两翼”中心城市建设。按照“循环发展引领、核心板块支撑、产业长廊带动、快速干道连接、优美小镇点缀、田园农业衬托、特色文化彰显”的思路，加快推进商州城区“北上东进”，推进丹凤县城“西融”和洛南县城“南移”，集中精力推进“一体两翼”中心城市建设，着力打造秦岭休闲之都、关天产业新城。今年编制完成“一体两翼”中心城市建设规划，以及土地利用、产业布局、交通发展三个专项规划，积极推进洛南、丹凤两县撤县设区工作。坚持“交通先行、做强主体、带动两翼、激活经济”的推进原则，加快“一体两翼”中心城市半小时交通环线建设。坚持“中疏外扩、成网增绿”的原则，实施城建重点工程65个，启动商运司区域旧城改造工程，全年完成中心城市建设投资40亿元以上。

二是加快城乡一体化发展步伐。支持商南、山阳、镇安、柞水四县县城建设，持续抓好沙河子省级重点示范镇建设，加大17个省、市重点镇建设力度，积极推进商镇“五位一体”及镇云等城乡统筹示范区建设，继续抓好新农村示范村建设，加快建设标准化新型农村社区。抓住国家加强中西部城市基础设施建设的机遇，加快城镇交通综合配套设施、地下综合管廊及污水垃圾处理设施建设。

三是有序推进农业转移人口市民化。放开县城和建制镇的户籍限制，有序放开中心城区落户限制，消除流动人口管理服务盲区。继续做好农村居民进城落户工作，整合城乡居民基本养老保险和基本医疗保险制度，鼓励农民工及早在城镇参保并连续参保；实行农民工与城镇居民同等的保障房分配制度，逐步将农民工子女纳入义务教育经费保障和普惠性学前教育、免费中等职业教育范围，完善符合条件的农民工随迁子女参加中考和高考政策；逐步理顺各级政府的财政分配关系，探索建立农业转移人口市民化成本分担机制。

四是全面提升城镇管理水平。积极推进经济社会发展、城镇建设和土地利用规划“三规合一”，增强规划实施的协调性和连续性。深入开展国家园林城市、生态示范城市、环保模范城市和卫生县城创建活动，积极推广应用现代信息技术，创新城市管理机制和方式，强化市容环境卫生综合整治，严厉查处各类违法建设行为，提高城市管理的精细化、智能化水平。

(六)创新管理，改善民生，提升人民群众的幸福指数

保障和改善民生是政府的首要责任。坚持财政支出和新增财力“两个80%”用于民生，促进民生持续改善、人民福祉不断提高。

一是进一步加强扶贫开发工作。认真实施秦巴山片区区域发展与扶贫攻坚规划，按照“实事求是、因地制宜、分类指导、精准扶贫”的要求，启动新一轮市级领导和市直部门包村扶贫工作，继续实施115个贫困村整村推进项目，确保8.4万贫困人口实现脱贫。

二是扎实抓好保障性住房建设。统筹实施保障性住房建设和棚户区改造，进一步加强保障性住房管理工作，全面推进廉租房与公共租赁住房并轨，在确保公平分配的同时提高入住率。全年新建各类保障性住房6183套，改造城市棚户区8773户。

三是深入推进陕南移民搬迁。坚持85%以上的集中安置率，加大公共配套设施建设和产业培育力度，认真研究解决移民搬迁中的突出问题，确保搬得出、稳得住、能致富。全年新建集中安置点116个，搬迁1.6万户、6.08万人。

四是大力实施就业优先战略。以高校毕业生、城镇困难人员、退役军人、农村转移劳动力为重点，继续做好基层医疗机构定向招聘本科毕业生工作，积极探索大学生创业孵化基地与创业贴息结合的政策，加强就业失业信息监测、技能培训和就业援助。全年新增城镇就业1.5万人，转移农村劳动力55万人，大学毕业生初次就业率保持85%以上。

五是健全完善社会保障体系。规范城镇职工医疗和生育保险市级统筹工作，完善社会保险关系转移接续政策。加强和规范城乡最低生活保障工作，加快推进城乡公益性公墓建设。完善社会养老和残疾人服务体系，做好计划生育特殊困难家庭扶助工作。加快区县儿童福利机构建设，大力发展妇女儿童事业和慈善事业，健全农村“三留守”人员关爱服务体系。

六是推动教育事业协调发展。坚持以“立德树人、深化素质教育、提升教育质量”为主线，继续做好“教育强县”和“双高双普”创建工作，大力推进义务教育均衡发展，加快普通高中多样化和职业教育适用型发展，加快市直职教资源整合，合理配置各类教育资源，逐步解决择校热、大班额等问题。继续实施“营养改善计划”，加强校车等学校安全管理。全面推进中小学校长任期目标考核制度，加强教师队伍建设，办好人民满意教育。

七是强化医疗卫生服务保障。围绕群众“少生病、方便看病、看得好病、看得起病”的总体目标，积极推进市区县镇医疗机构综合改革，健全推广重大疾病医疗救助制度，继续完善药品“三统一”补偿政策，在市级医疗机构建立药品质量可追溯制度，逐步提高城镇居民和新农合人均政府补助标准。积极推进“三万”工程，鼓励社会力量办医，加快发展中医药事业。坚持计划生育基本国策，制定落实“单独二孩”有关政策。

八是加快发展文化体育事业。抓好市广播电视演播大厅建设，完成重点镇综合文化中心和广播电视户户通、农村应急广播网建设任务，做好文化设施设备配送和“农家书屋”管理使用工作，广泛开展群众文化和全民阅读活动。加强文化遗产保护，深入开展“扫黄打非”，培育文化市场主体，积极推进政府购买公共演出工作，力争文化产业增长27%以上。加快建设市县公共体育场馆和镇村体育健身场所，确保市体育场建成投入使用。

九是全力维护社会大局稳定。围绕建设法治商洛、平安商洛，做好安全生产和平安生活各项工作。严格落实安全生产责任制，推进政府监管网格化、企业管理精细化、行业直管专业化，深入开展重点行业领域专项整治，有效防范各类安全事故发生。加强食品药品监管能力建设，做好食品放心区县创建和药品安全责任体系评价工作。加快市公安监管中心建设，构筑立体化社会治安防控体系，严密防范、严厉打击各类违法犯罪活动，不断增强人民群众的安全感。健全重大决策社会稳定风险评估和突发事件应急管理机制，完善领导干部接访下访和包案长效机制，加大各类矛盾纠纷排查调处力度，依法保护被征地拆迁群众的合法权益。继续加强民族宗教工作，探索建立新型社区管理体制。

(七)保护环境，建养生态，提升可持续发展能力

坚持绿色、低碳、循环、清洁的发展理念，突出重点行业和领域，强化科学治理，加大源头保护，严守生态红线，狠抓节能减排，守住青山绿水，留住蓝天白云，建设生态文明。

一是大力推进循环经济发展。加强国家循环经济示范城市创建工作，大力推进循环发展和综合利用，拓展完善10条循环产业链，构建完整的循环产业体系。加快28个循环经济和尾矿资源综合利用项目建设，认证资源综合利用企业和循环经济试点企业10户，支持商丹园区创建国家循环化改造示范园区。大力推广循环农业发展模式，实现资源高效利用和循环使用。

二是切实抓好丹江等流域污染防治。围绕“一江清水供京津”的政治任务，全面推进丹江等流域污染防治三年行动计划，切实抓好重金属、危险化学品和危险废物污染防治，加快推进企业中水利用和丹江湿地公园建设，突出解决丹江沿线垃圾污水处理厂建设和正常运营问题。加强环境监测和应急处置能力建设，严格落实丹江断面监测月考核、季评比和定期公布制度，确保丹江出境断面水质全面达标。

三是加强农村清洁和大气污染防治。以“清洁水源、清洁家园、清洁田园、清洁能源”为目标，继续加强农村环境综合整治，着力解决农业面源污染等问题。认真落实“治污降霾”国十条和《陕西省大气污染防治条例》，加强对锅炉燃煤、机动车尾气、建筑扬尘和挥发性有机物污染的监管和限制，从源头上防治大气污染，确保空气质量持续改善。

四是扎实做好节能降耗工作。全面推进工业、建筑、交通和公共机构节能，实施好14个节能技改项目，重点抓好30家耗能大户的节能降耗，坚决淘汰落后产能，促进传统产业升级。把节约用地贯穿经济社会发展的全过程，切实提高土地利用效率。加快高耗水工业技术改造，实现水资源集约高效利用。

五是加大生态环境建设力度。按照陕南山地森林化的要求，抓好林业重点工程建设，大力发展林下产业，扩大退耕还林成果。实施城周重点区域绿化三年规划，加快推进城市“绿道”建设，加大公路两边生态建设和绿化力度，全年完成造林绿化31万亩。强力推进丹江流域综合治理，完成20条小流域综合治理任务，治理水土流失700平方公里。

(八)深化改革，扩大开放，增强经济社会发展活力

认真贯彻落实中央全面深化改革的决定和省委实施意见，积极稳妥推进重点领域改革，全面加快对外开放步伐，进一步增强发展的内生动力。

一是积极推动行政体制改革。加快推进政府职能转变和简政放权，强化市场监管和便民服务职能。做好省上下放行政审批事项承接工作，分批取消和下放部分市级审批事项，规范工作流程，简化办事程序，提高工作效率。积极推进市区县镇政府机构改革，做好卫生和计生机构整合工作，深入推进事业单位分类改革。严格控制机构和编制，确保财政供养人员只减不增。

二是深入推进经济体制改革。全面落实工商注册制度便利化政策，继续清理行政事业性收费，探索建立城乡统一的建设用地市场，加大招拍挂方式出让探矿权力度，营造公平竞争的市场秩序。积极推进“营改增”工作，完善镇办财政管理体制，规范政府债务管理，健全风险预警机制。深化收入分配制度改革，规范收入分配秩序。继续完善融资担保体系，鼓励民间资本设立中小银行。

三是大力发展非公有制经济。制定加快非公有制经济发展的政策措施，积极发展混合所有制经济，加大小微企业支持力度，年内新增私营企业600户、个体经营户6000户，非公有制经济增加值占GDP的比重保持50%以上。加快商丹园区省级出口综合示范基地建设，支持商南县创建省级专业型出口示范基地，确保全市外贸进出口总额增长11%。

四是深度开展招商引资工作。开展客商评议职能部门和科(股)长活动，完善社会信用体系，切实优化发展环境。依托园区招商平台，创新招商引资理念，采取以商招商、商会招商和专题招商等形式，重点抓好现代材料、旅游、农产、医药和水产业招商，切实强化招商引资的针对性和项目跟进服务工作，力争招商引资到位资金350亿元以上。

四、努力建设人民满意的政府

深化发展的新要求和人民群众的新期待，对政府自身建设提出了新的更高要求。我们必须紧抓发展这个第一要务，切实强化担当意识，增强担当勇气，提升担当能力，始终带着感情、付出真情、充满激情，竭尽全力把党和人民赋予的职责履行好，努力打造敢担当、有作为的政府。

我们要解放思想、大胆创新。从发展大局出发，加强形势研判，把差距视作潜力，把压力化为动力，善于借“外力”，敢于闯“难关”，用魄力去开拓，用智慧去创造，主动作为，奋勇争先，做改革创新的推动者和实践者。

我们要科学决策、依法行政。更加自觉地接受市人大及其常委会的监督和市政协的监督，进一步完善联系人大代表和政协委员制度。切实规范行政执法程序，真正做到公正文明执法。全面推进政务公开，建立健全百姓问政制度，做好网上诉求办理和政务微博发布，加快推进决策公开、管理公开、服务公开、结果公开，努力做到让人民监督权力、让权力在阳光下运行。

我们要改进作风、务实为民。深入开展群众路线教育实践活动，市政府班子成员坚持以身作则，切实解决群众反映的突出问题。严格执行八项规定和党政机关厉行节约反对浪费条例，全面推进部门预决算和“三公”经费公开，坚决停止新建楼堂馆所，坚决清理违规使用办公用房，加快推进公车改革，加强因公出国管理，强化机关事务管理，规范公务接待活动，切实改进文风会风，努力降低行政成本。

我们要清廉自律、廉政勤政。严格落实党风廉政建设责任制，健全完善廉政风险防控体系，强化效能监察、执法监察和审计监督，加大违纪案件查办和政风行风整治力度，着力整治公务人员庸懒散浮等问题，树立廉政勤政的良好政府形象。

我们要完善机制、强化落实。开展“政府执行力提升年”活动，以“踏石有印、抓铁留痕”的决心，改进“抓”的方法，树立“干”的导向，大力推行“一线工作法”和现场办公，加强督查考评，强化行政问责，确保各项工作快速顺利推进。

各位代表，长期以来，驻商部队和武警官兵为商洛做出了重要贡献，特别是在承担急难险重任务中发挥了突击队作用，全市人民永远铭记在心。我们将一如既往地支持驻商部队革命化现代化正规化建设，继续加强国防教育、国防动员和民兵预备役力量建设，加快推进武警和消防队站及人民防空建设，不断巩固和发展军政军民团结，努力开创双拥共建工作的新局面！

各位代表，人民群众对美好生活的向往是我们的奋斗目标，全市人民生活得更加幸福是我们的不懈追求，加快推进商洛发展需要全市人民的共同努力。让我们紧密团结在以习近平同志为总书记的党中央周围，高举中国特色社会主义伟大旗帜，在市委的坚强领导下，抢抓机遇，开拓进取，团结实干，克难攻坚，为加快建设幸福商洛做出新的更大的贡献！
